# Impact of corticosteroids on the duration of ventilatory support during severe acute exacerbations of chronic obstructive pulmonary disease in patients in the intensive care unit: a study protocol for a multicentre, randomized, placebo-controlled, double-blind trial

**DOI:** 10.1186/s13063-023-07229-9

**Published:** 2023-03-26

**Authors:** Alexis Ferré, Arnaud W. Thille, Armand Mekontso-Dessap, Thomas Similowski, Stéphane Legriel, Philippe Aegerter, Alexandre Demoule, The Réseau Européen de Recherche en Ventilation Artificielle (REVA) research network

**Affiliations:** 1 Intensive Care Unit, Versailles Hospital, Le Chesnay, France; 2 Service de Médecine Intensive Réanimation, CHU de Poitiers, Université de Poitiers, Poitiers, France; 3 Service de Médecine Intensive Réanimation, APHP. Hôpitaux Universitaires Henri Mondor, Créteil, France; 4 Département R3S, APHP. Sorbonne Université, Hôpital Pitié-Salpêtrière, Paris, France; 5 Sorbonne Université, Inserm, UMRS1158, Paris, France; 6 University Paris-Saclay, UVSQ, INSERM, CESP, Team “PsyDev”, Villejuif, France; 7 Groupement Inter-Régional de Recherche Clinique Et d’Innovation (GIRCI) – Île-de-France, Cellule méthodologique – Santé Publique UVSQ-Inserm U1168, Paris, France; 8 Service de Médecine Intensive Réanimation, APHP. Sorbonne Université, Hôpital Pitié- Salpêtrière, Paris, France

**Keywords:** Corticosteroids, Exacerbation, COPD, Mechanical ventilation, Mortality

## Abstract

**Background:**

Patients who are admitted to the intensive care unit (ICU) for severe acute exacerbations of chronic obstructive pulmonary disease (COPD) have poor outcomes. Although international clinical practice guidelines cautiously recommend the routine use of systemic corticosteroids for COPD exacerbations, data are scarce and inconclusive regarding their benefit for most severe patients who require mechanical ventilation in the ICU. Furthermore, corticosteroids may be associated with an increased risk of infection, ICU-acquired limb weakness, and metabolic disorders.

**Methods and analysis:**

This study is an investigator-initiated, multicentre, randomized, placebo-controlled, double-blind trial comparing systemic corticosteroids to placebo during severe acute exacerbations of COPD in patients who require mechanical ventilation in French ICUs. A total of 440 patients will be randomized 1:1 to methylprednisolone (1 mg/kg) or placebo for 5 days, and stratified according to initial mechanical ventilation (non-invasive or invasive), pneumonia as triggering factor, and recent use of systemic corticosteroids (< 48 h). The primary outcome is the number of ventilator-free days at day 28, defined as the number of days alive and without mechanical invasive and/or non-invasive ventilation between randomization and day 28. Secondary outcomes include non-invasive ventilation (NIV) failure rate, duration of mechanical ventilation (invasive and/or NIV), circulatory support (vasopressor), outcomes related to corticosteroid adverse events (severe hyperglycaemia, gastrointestinal bleeding, uncontrolled arterial hypertension, ICU-acquired weakness, ICU-acquired infections, and delirium), lengths of ICU and hospital stay, ICU and hospital mortality, day 28 and day 90 mortality, number of new exacerbation(s)/hospitalization(s) between hospital discharge and day 90, and dyspnoea and comfort at randomization, ICU discharge, and day 90. Subgroup analyses for the primary outcome are planned according to stratification criteria at randomization.

## Administrative information

Note: the numbers in curly brackets in this protocol refer to SPIRIT checklist item numbers. The order of the items has been modified to group similar items (https://www.equator-network.org/reporting-guidelines/spirit-2013-statement-defining-standard-protocol-items-for-clinical-trials/)

**Table:** 

Title {1}	Impact of corticosteroids on the duration of ventilatory support during severe acute exacerbations of chronic obstructive pulmonary disease in patients in the intensive care unit: a study protocol for a multicentre, randomized, placebo-controlled, double-blind trial
Trial registration {2a and 2b}	ClinicalTrials.gov Identifier: NCT04163536. Registration date: November, 14, 2019 https://clinicaltrials.gov/ct2/show/NCT04163536
Protocol version {3}	The current version of the protocol is Version 5.0; November, 3, 2022.
Funding {4}	The study was funded by grant from the French Ministry of Health obtained in 2018 (Programme Hospitalier de Recherche Clinique): PHRC-18–0127
Author details {5a}	-Alexis Ferré, Intensive Care Unit, Versailles Hospital, Le Chesnay, France. -Arnaud W. Thille, Service de Médecine Intensive Réanimation, CHU de Poitiers, Université de Poitiers, Poitiers, France -Armand Mekontso-Dessap, Service de Médecine Intensive Réanimation, APHP. Hôpitaux Universitaires Henri Mondor, Créteil, France -Thomas Similowski, Département R3S, APHP. Sorbonne Université, Hôpital Pitié- Salpêtrière, Paris, France; Sorbonne Université, Inserm, UMRS1158, Paris, France. -Stéphane Legriel, Intensive Care Unit, Versailles Hospital, Le Chesnay, France; University Paris-Saclay, UVSQ, INSERM, CESP, Team "PsyDev", Villejuif, France. -Philippe Aegerter, Groupement Inter-régional de Recherche Clinique et d’Innovation (GIRCI) – Île-de-France, Cellule méthodologique – Santé Publique UVSQ-Inserm U1168, France. -Alexandre Demoule, Service de Médecine Intensive Réanimation, APHP. Sorbonne Université, Hôpital Pitié- Salpêtrière, Paris, France.
Name and contact information for the trial sponsor {5b}	Laure MORISSET and Virginie CHATAGNER Délégation à la recherche clinique et à l’innovation (DRCI) Centre hospitalier de Versailles (CHV) 177 rue de Versailles, 78,150, Le Chesnay, France Phone: + 33–1-39–23-97–85
Role of sponsor {5c}	The sponsor has no input in the study design, protocol preparation, or future data analysis and interpretation.

## Strengths and limitations of the study

- ▶ No large study has yet shown a beneficial effect of corticosteroids on patient outcomes in severe acute exacerbations of chronic obstructive pulmonary disease (COPD).
- ▶ This large randomized, placebo-controlled trial may help to establish strong recommendations on corticosteroid use in patients admitted to the intensive care unit (ICU) for severe acute exacerbations of COPD with a high level of evidence.
- ▶ Whereas open-label trials have previously been performed, this is a double-blind study comparing corticosteroids versus placebo.
- ▶ The sample size of this trial has been designed to have the power to show an absolute reduction of ventilator-free days at day 28.

## Introduction

### Background and rationale {6a}

Chronic obstructive pulmonary disease (COPD) is the third leading cause of mortality worldwide, resulting in over 3 million deaths in 2019 [1]. In patients with COPD, a severe acute exacerbation is defined as respiratory failure with hypercapnic acidosis requiring mechanical invasive and/or non-invasive ventilation, which occurs in around 20% of exacerbations [2]. Despite improving intensive care unit (ICU) management, severe acute exacerbations are associated with a high mortality rate (15% in the ICU and 20% at hospital discharge), making it a life-threatening event [3]. Treatments that can improve this outcome are obviously of great medical and socioeconomic interest.

The goal for COPD exacerbation treatment is to reduce the negative impact of the current exacerbation and to prevent subsequent events. As COPD exacerbations are associated with general and lung inflammation, systemic corticosteroids have been evaluated in this field. Several studies have shown that systemic corticosteroids may be beneficial in patients admitted to the ward for non-critical COPD exacerbations [4–9]. In such patients, corticosteroids could reduce treatment failure, hospital length of stay, and early relapse, but they have no impact on mortality [10]. However, most of these studies have excluded critically ill patients with COPD.

To date, only two studies have examined the impact of corticosteroids in patients with COPD admitted to the ICU [11, 12]. One of these showed a benefit of corticosteroids on NIV failure rate and duration of mechanical ventilation [11], while the other did not confirm these results [12]. However, these two studies were underpowered (*n* = 87 and *n* = 217, respectively) with inclusion rates that were lower than expected due to the stringent exclusion criteria and a limited number of participating centres. They were both ended before completion of the planned sample size. Currently, the potential benefit of systemic corticosteroids in patients with COPD admitted to the ICU for severe acute exacerbations has not been confirmed, which does not allow for strong recommendations [13].

The hypothesis of the present study is that, compared to placebo, corticosteroids would hasten respiratory recovery and subsequently decrease the duration of mechanical ventilation (invasive and/or NIV) and mortality in patients admitted to the ICU for severe acute exacerbations of COPD.

### Objectives {7}

The primary objective is to compare the effect of systemic corticosteroids versus placebo on the number of ventilator-free days during the 28 days after randomization in patients with COPD admitted to an ICU, a step-up unit, or a respiratory care unit for a severe acute exacerbation requiring mechanical ventilation (invasive and/or NIV).

The secondary objectives are to compare the effect of systemic corticosteroids versus placebo on:

- The rates of NIV failure (defined as the need for intubation within the 7 days following randomization)
- The duration of mechanical ventilation (invasive and NIV)
- Circulatory support (use of vasopressor) within 28 days of randomization
- Lengths of stay in the ICU/step-down unit/respiratory care unit and in the hospital
- Dyspnoea and comfort (patient-reported outcome) at randomization, ICU discharge, and day 90
- ICU, in-hospital, and day 28 and day 90 mortalities, as well as the standardized mortality ratio
- The proportion of new exacerbations/hospital admissions between hospital discharge and day 90
- The proportion of patients with adverse events related to corticosteroids (including severe hyperglycaemia during the first 5 days after randomization, gastrointestinal bleeding, uncontrolled arterial hypertension, ICU-acquired weakness, ICU-acquired infections, and delirium).

### Trial design {8}

CORTICOP is an investigator-initiated, phase 3, multicentre, parallel-group, double-blind, randomized (1:1), placebo-controlled trial comparing systemic corticosteroids versus placebo in patients admitted to an ICU for a severe acute exacerbation of COPD who require mechanical ventilation (invasive or NIV). The trial will be done in accordance with the Standard Protocol Items: Recommendations for Interventional Trial guidelines.

## Methods: participants, interventions and outcomes

### Study setting {9}

**Fig. 1:**
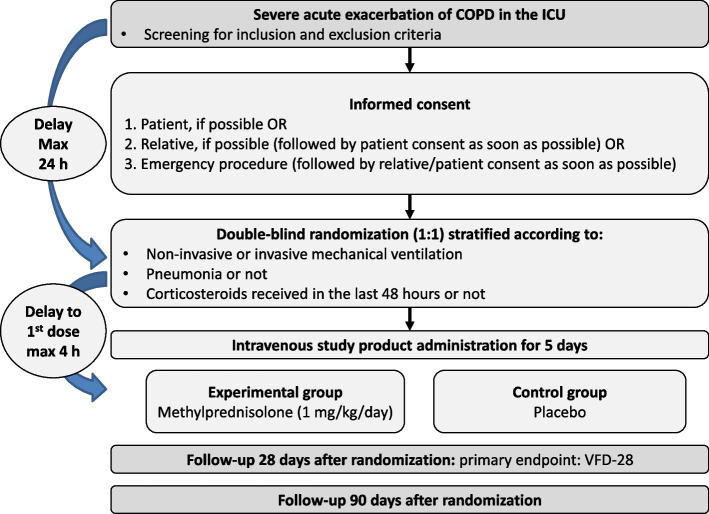
Study design. COPD: chronic obstructive pulmonary disease; ICU: intensive care unit; VFD-28: ventilator-free days and alive at day 28

This study will be conducted in 24 ICUs in France. The study design is outlined in Fig. 1, while Table 1 details the schedule of assessments and procedures.

**Table 1:** Schedule of assessments and procedures

	**Day 0**	**Daily (days 1–27)**	**Day 28 or ICU discharge**	**Day 90**^**a**^
Inclusion and exclusion criteria	X			
Informed consent	X			
Randomization (between day 0 and day 1)	X			
Demographic data and comorbidities	X			
Clinical data	X	X	X	
Laboratory data (as appropriate)	X	X	X	
Eosinophilic polynuclear cell count in blood (before 1st dose of protocol treatment) if data available	X			
Radiological data (within 24 h)	X			
Microbiological data (within 48 h)	X			
SAPS II	X			
SOFA (if data available)	X	Days 3, 7, 14, 21	X	
RASS (if invasive mechanical ventilation)	X	X	X	
Mechanical ventilation	X	X	X	
NIV (duration/day, IP, PEEP, FiO_2_)	X	X	X	
Invasive MV (mode, IP or RR × tidal volume, PEEP, FiO_2_)	X	X	X	
Sedative drugs	X	X	X	
Neuromuscular blockers	X	X	X	
Catecholamine infusion	X	X	X	
Adverse event potentially related to corticosteroids and/or changing study drug		X	X	
Serious adverse event^b^	X	X	X^b^	X^c^
ICU length of stay		X	X	X
Hospital length of stay		X	X	X
Number of new exacerbation(s)/hospitalization(s) since hospital discharge				X
Dyspnoea, comfort (patient-reported outcome)	X		X	X
Vital status		X	X	X

*SAPS II* Simplified Acute Physiology Score II, *SOFA* Sepsis Organ Failure Assessment, *RASS* Richmond Agitation-Sedation scale, *NIV* Non-invasive ventilation, *IP* Inspiratory pressure, *PEEP* Positive end-expiratory pressure, *FiO*_*2*_ Fraction of inspired oxygen, *MV* Mechanical ventilation, *RR* Respiratory rate, *ICU* Intensive care unit

^a^Telephone call

^b^Serious adverse events will be collected for 20 days after the last experimental treatment injection

^c^Only if the serious adverse event is related to the study medication

### Eligibility criteria {10}

Inclusion criteria are the following:

- Age ≥ 40 years
- Strongly suspected or documented COPD, defined by the presence of the following criteria: persistent respiratory symptoms (dyspnoea, chronic cough, or sputum production), history of exposure to a risk factor (e.g. tobacco smoke), pulmonary function tests (if available) showing airflow limitation not fully reversible (post-bronchodilator ratio of forced expiratory volume in 1 s [FEV_1_] to forced vital capacity [FVC] < 0.7)
- Severe acute exacerbation, defined by the presence of:
  - COPD exacerbation, defined as a change in baseline respiratory symptoms (dyspnoea, cough, or sputum) requiring a change in regular respiratory medication AND
  - Acute respiratory failure (defined as clinical signs of excessive muscle activity, polypnea ≥ 30 breaths/min, or the use of accessory respiratory muscles) requiring mechanical ventilation (possibly already started within the previous 24 h), either invasive or NIV (implemented due to hypercapnic acidosis with partial pressure of carbon dioxide [PaCO_2_] ≥ 45 mmHg and pH ≤ 7.35)
- Admission to an ICU, a step-up unit, or a respiratory care unit within the last 24 h
- Written informed consent from the patient or their surrogates. For patients who are not able to consent at admission (e.g. due to hypercapnic encephalopathy) and in absence of a substitute decision-maker, an emergency inclusion procedure will be allowed, with a mandatory delayed consent
- Affiliation to (or benefit from) the French health insurance system

Exclusion criteria are the following:

- Previous diagnosis of asthma according to the “Global initiative for Asthma” international guidelines
- Current and proven SARS-CoV2 infection
- Contraindication to systemic corticosteroid treatment: allergy to corticosteroids, uncontrolled severe arterial hypertension, uncontrolled diabetes mellitus, gastrointestinal ulcer bleeding
- Underlying disease requiring chronic daily use of systemic corticosteroids
- Pneumothorax at inclusion
- Extracorporeal life support (ECMO or ECCO2R) at inclusion
- Moribund patient life expectancy < 3 months
- Pregnancy
- Patients protected by law (under guardianship or curatorship)
- Exclusion period linked to enrolment in another interventional clinical trial that could influence the primary outcome
- Previous inclusion in the current study

### Who will take informed consent? {26a}

Patients will only be included after proving written informed consent to the investigator. If the patient is not able to understand the information given, they can be included if the same procedure is completed with their next of kin. Patients who are unable to provide informed consent and for whom a substitute decision-maker is not present may be included through a process of deferred consent. After the patient’s recovery, they will be asked if they agree to continue in the trial. The protocol has been approved by an independent Ethics Committee (Comité de Protection des Personnes Ouest VI, Brest, France).

### Additional consent provisions for collection and use of participant data and biological specimens {26b}

Not applicable, no samples were collected.

## Interventions

### Explanation for the choice of comparators {6b}

Methylprednisolone is a corticosteroid that has been previously used in a study evaluating steroids in severe acute exacerbation of COPD [11], and which can be administered intravenously. The placebo arm will act as a control group for the methylprednisolone arm.

### Intervention description {11a}

Randomization should be performed no later than 24 h after all inclusion and exclusion criteria have been completed.

Study drug (corticosteroid or placebo) should be administered no later than 4 h after randomization. Instructions for the reconstitution of the study drug are provided on a leaflet in the treatment kit.

In the experimental group, methylprednisolone will be administered intravenously for 5 days at a dose of 1 mg/kg/day. Methylprednisolone will be provided for reconstitution and dilution in 50 mL of NaCl 0.9% solution and administered over 15 min, once a day.

In the control group, placebo will be administered intravenously for 5 days. The placebo will be identical in appearance to the experimental substance. Placebo will be provided as 50 mL of NaCl 0.9% solution and administered intravenously over 15 min, once a day.

### Criteria for discontinuing or modifying allocated interventions {11b}

In case of any serious adverse events during study treatment, if necessary, the prespecified unblinding procedure could be initiated. The local investigator, the promotor, or the pharmacy could initiate the unblinding procedure with a secret code dedicated for each treatment container.

Apart from protocol study treatment, systemic corticosteroids are strongly discouraged during the study period, especially from day 1 to day 5. However, in case of septic shock requiring vasopressors, laryngeal oedema identified just before weaning from mechanical ventilation, and/or severe worsening bronchospasm despite optimal treatment, rescue corticosteroids could be initiated according to the attending physician’s decision. Rescue corticosteroids will be reported in the electronic case report form (eCRF).

### Strategies to improve adherence to interventions {11c}

This study will include patients in the ICU who have been admitted in a critical condition and are under continuous medical supervision. Thus, administration and adherence to the protocol treatment will be closely monitored during the hospital stay.

### Relevant concomitant care permitted or prohibited during the trial {11d}

All standard practices will be continued throughout the study period.

In accordance with international guidelines for patients with COPD, NIV will be used as first-line therapy for signs of respiratory failure with respiratory acidosis, e.g. pH ≤ 7.35 and hypercapnia with PaCO_2_ ≥ 45 mmHg [14]. For NIV use, there are no pre-established settings in current guidelines. Nonetheless, the study protocol includes standardization to minimize the effect of current inhomogeneous practices on the primary and secondary endpoints. For this purpose, an indicative NIV algorithm is detailed in the study protocol.

NIV failures that require invasive mechanical ventilation will be defined as previously reported in studies using NIV [2, 14–17] and correspond to current clinical daily practice in the ICU. Finally, the formal indications for invasive mechanical ventilation will be defined in a standardized manner, such as (i) NIV failure (neurological worsening with persistent hypercapnic acidosis; respiratory and hypercapnic acidosis worsening; refractory hypoxemia under NIV); (ii) cardiac arrhythmias leading to circulatory failure; (iii) shock requiring a vasopressor; (iv) cardiopulmonary arrest; or (v) respiratory failure and hypercapnic acidosis with contraindication to NIV (surgery, deformity or facial trauma, or digestive intolerance with uncontrollable vomiting making NIV impossible). For invasive mechanical ventilation, the procedure and ventilatory settings will be standardized according to international recommendations. In case of acute respiratory distress syndrome (ARDS) (Berlin definition [18]), settings of ventilatory parameters will be left to the discretion of the clinician in charge but in accordance with international ARDS guidelines.

In case of successful NIV (without invasive mechanical ventilation), the weaning process will be progressive. In the absence of universal established criteria, the NIV withdrawal procedure will be standardized but with flexibility left to the clinician in charge of the patient. The weaning will take clinical and gasometrical criteria into account, but will only start after a minimum of 24 h of NIV. If the strategy of reducing the number of NIV sessions is successful after a 24-h period, the weaning period will start. In case of poor tolerance of the weaning process (clinical and/or gasometrical), NIV sessions will be increased by 50% until stabilization (no sign of acute respiratory failure, breath rate < 30/min with stable pH > 7.35, and normocapnia or mild stable hypercapnia [< 50 mmHg]).

During the weaning process of invasive mechanical ventilation, patients fulfilling the recommended prespecified criteria must be given a spontaneous breathing trial [17, 19]. The modalities of this test will be chosen according to the practices of each centre (minimum ventilatory assistance or “T” tube test). The extubation decision will be left to the clinician’s decision according to international guidelines.

After weaning from invasive mechanical ventilation, prophylactic NIV will be used for all extubated patients because underlying COPD puts patients at high risk of extubation failure [14, 20]. NIV will be stopped after 24 h according to clinical respiratory status associated with stable arterial blood gas. For patients with persistent moderate to severe respiratory symptoms, hypoxaemia (defined as a need for FiO_2_ ≥ 50% to maintain SpO_2_ ≥ 92% or PaO_2_/FiO_2_ ≤ 150 mmHg) and/or hypercapnic acidosis (pH ≤ 7.35 with PaCO_2_ ≥ 45 mmHg), NIV should be continued until clinical and/or gasometrical improvement for 24-h periods until complete resolution. The rhythm and duration of NIV during each 24-h period will be left to the clinician’s discretion.

### Provisions for post-trial care {30}

Ancillary and post-trial care are not planned. However, serious adverse events will be collected during the 20 days after the last experimental treatment injection. If an adverse event occurs, surveillance will be strengthened and a symptomatic product can be added.

For the duration of the study, the Versailles hospital (sponsor) has taken out an insurance policy with the SHAM Company, guaranteeing its own third-party liability as well as the third-party liability of any intervener (medical doctors or personnel involved in carrying out the research) according to Article L.1121–10 of the Code de la Santé Publique (CSP; French Public Health Code). It will also ensure full compensation for any harmful consequences of the research to the affected person or their beneficiaries, unless proven that the damage is not attributable to the study or any intervener related to the study.

### Outcomes {12}

#### Primary outcome measure

Number of ventilator-free days (VFDs) at day 28 is defined as the number of days alive without mechanical ventilation (invasive and/or NIV) between randomization and day 28.

VFDs are defined as follows:

- VFDs = 0 if the patient dies before 28 days or the patient requires mechanical ventilation for ≥ 28 days
- VFDs = 28 − x if the patient is successfully weaned from mechanical ventilation within 28 days, where *x* is the number of days spent receiving mechanical ventilation (invasive and/or NIV).

#### Secondary outcome measures

- NIV failure rate, defined as the need for intubation within the 7 days following randomization
- The duration of invasive mechanical ventilation
- The total duration of mechanical ventilation (including invasive and NIV)
- Vasopressor-free days and alive at day 28
- Length of stay in the ICU, the step-down unit, and the respiratory care unit
- In-hospital length of stay
- Dyspnoea and comfort (patient-reported outcome) at inclusion, ICU discharge, and day 90 assessed using the dyspnoea analogue scale: “How much is the intensity of the worst breathing difficulty you have experienced in the last 24 h” from 0 (none) to 10 (extremely); “How much anxiety/distress has the breathing difficulty caused you in the last 24 h” from 0 (none) to 10 (extremely)
- Dyspnoea and comfort (patient-reported outcome) with standardized questionnaires at day 90: COPD assessment test and the Dyspnoea-12 questionnaire
- Number of new exacerbation(s)/hospitalization(s) between hospital discharge and day 90
- ICU, in-hospital, day-28, and day-90 mortalities
- Standardized mortality ratio
- Number of severe hyperglycaemias requiring intravenous insulin therapy during the first 5 days
- Number of gastrointestinal bleeding(s), defined as an acute loss of 2 g/dL of haemoglobin requiring a red blood cell transfusion or gastroscopic evaluation
- Number of uncontrolled arterial hypertension(s): unusual hypertension requiring the introduction or addition of an antihypertensive medication compared to usual medications
- ICU-acquired weakness (Medical Research Council [MRC] score < 48/60) assessed on day 28 or at the time of ICU discharge
- Number of ICU-acquired infections: bacteraemia of any cause, catheter-related infection, hospital-acquired pneumonia (non-intubated patients), ventilator-associated event (intubated patients), probable or possible ventilator-associated pneumonia
- Delirium during the ICU stay.

### Participant timeline {13}

The participant timeline is detailed in Table 1.

### Sample size {14}

Based on a large database that included 35 ICUs [3], the mortality rate associated with severe acute exacerbations of COPD is 17% and the mean duration of ventilation (invasive or NIV) is 5.8 days for alive patients (median 4 days). According to this database and previous published studies, 75% of patients are treated with NIV, while 25% are treated with invasive mechanical ventilation. We performed several simulations in which we varied the mortality rate (binomial, 15–19%) and the mean duration of ventilation (log-normal distribution, 5–7 days) and calculated the required sample size according to alpha risk = 5% and beta risk = 20% and various hypotheses of efficacy (none or 1% reduction in the mortality rate, combined with a 2–4-day reduction in days of ventilation) and a Wilcoxon test of the resulting VFD in the two groups. It showed that 200 patients per group would allow the detection of at least a 2-day reduction in ventilation regardless of the impact on mortality. Accounting for withdrawal of consent, a 10% increase was allowed, i.e. a sample size of 220 per group, for a total of 440.

### Recruitment {15}

Consecutive patients will be recruited in the ICU by the investigators.

## Assignment of intervention: allocation

### Sequence generation {16a}

The randomization list will be prepared by the clinical trial research unit of the Versailles hospital. The randomization ratio will be 1:1. The randomization list will be computer generated with random permuted blocks. Randomization will be performed after inclusion of the patient on the eCRF (Posicube© for Cleanweb© website administration).

Randomization will be stratified on: (i) initial mechanical ventilation (NIV or invasive mechanical ventilation), as the risk of death is markedly increased in patients who require invasive mechanical ventilation; (ii) pneumonia (yes/no), as this triggering factor may impact on the potential benefits of corticosteroids; and (iii) corticosteroid administration in the last 48 h (yes/no) at the time of randomization. Randomization will be carried out by connecting to the centralized eCRF website.

### Concealment mechanism {16b}

Treatment administration will be done according to the randomization arm following the pre-established randomization list.

Sequentially numbered containers of methylprednisolone or placebo with identical appearance prepared by the pharmacy will be stored in the ICU. The computer randomization procedure will generate a container (kit box) number. The available container with the computer-generated number in the department’s stock will be assigned to the newly included patient in order to proceed with the randomization.

### Implementation {16c}

The randomization sequence of methylprednisolone versus placebo will be generated on the eCRF. The group assignment will then be provided to the investigator and the study coordinator.

## Assignment of interventions: blinding

### Who will be blinded {17a}

Trial participants, physicians in charge, nurses in charge, researchers involved in the study, and outcome assessors will be blinded.

### Procedure for unblinding if needed {17b}

Unblinding will be permissible in case of severe adverse events.

Instances that require immediate unblinding will be handled according to the Versailles hospital standard operation procedures. For this blinded trial, an emergency procedure will be applied. Each pharmacy and/or local investigator will have an unblinding card for emergency management. The reason for breaking the code must be documented in the source document and on the eCRF page, along with the date and the initials of the person who broke the code. The sponsor must be informed as soon as possible of the unblinding by the centre.

## Data collection and management

### Plans for assessment and collection of outcomes {18a}

The investigator and the study coordinator will be responsible for data collection.

### Plans to promote participant retention and complete follow-up {18b}

No participants will be lost to follow-up providing they remain in the hospital. Before hospital discharge, patient information, including their telephone number and those of their relatives will be noted in their personal medical file to ensure the completion of follow-up at day 90.

### Data management {19}

Designated staff will enter the data into the eCRF under the supervision of the principal investigator in each centre, who will be responsible for ensuring that the data collected are complete, accurate, and that entry is performed in a timely manner. A data manager will be in charge of checking missing or inconsistent data, and providing queries to be solved. In case of missing data, the reason will be noted. Corrections, with the reason for the corrections, will also be recorded/tracked in the eCRF.

After resolution of all queries, the database will be locked for statistical analysis.

### Confidentiality {27}

Participants included in this research will be identified using a unique anonymous reference number that will be used for the entire duration of the study. For eligible patients who are not included in the study, the date and reason for non-inclusion will be anonymously collected in order to build a patient flow chart.

All study-related information will be stored securely at the study site. All participant information will be stored in locked filing cabinets in areas with limited access. All reports, data collection, processes, and administrative forms will be identified only by a coded identification number to maintain participant confidentiality. All records that contain names or other personal identifiers, such as locator forms and informed consent forms, will be stored separately from study records identified by code number.

Data will be handled according to French law on data protection and European General Data Protection Regulation (GDPR). All original records will be archived at trial sites for 15 years.

### Plans for collection, laboratory evaluation and storage of biological specimens for genetic or molecular analysis in this trial/future use {33}

Not applicable, as no specimens will be collected.

## Statistical methods

### Statistical methods for primary and secondary outcomes {20a}

All analyses will be performed by the study statistician according to a predefined statistical analysis plan, using the latest versions of R software© (R Foundation for Statistical Computing, Vienna, Austria) or Statistical Analysis System© (SAS). A two-tailed *p* value of less than 0.05 will be considered as indicating statistical significance.

In accordance with the CONsolidated Standards Of Reporting Trials (CONSORT) statement, a flow diagram will describe the progress of patients in the two groups through the phases of the trial (enrolment, intervention allocation, intervention received, follow-up and data analysis). The analysis will be performed on an intention-to-treat basis. In case of premature stop or withdrawal from the study, patients will not be substituted. Missing values will be described and, according to nature and frequency, multiple imputation methods will be used. A per-protocol analysis will be used as a sensitivity analysis, excluding patients who were wrongly randomized or did not receive the allocated intervention. Comparative analysis will systematically be done with (main analysis) and without adjustment on randomization stratification factors.

Quantitative data will be described as means, medians, standard deviations, and interquartile ranges. Nominal data will be described as percentages with 95% confidence intervals. Proportions of missing data will be given. Patient characteristics will be described by centre and compared. Quantitative data will be compared using *t*-tests or non-parametric tests (Mann–Whitney sum rank) when the distribution does not approach symmetry even after suitable transformation (Tukey’s ladder of powers). Qualitative data will be compared by means of chi-square tests or Fisher exact tests. Time-to-event data (survival) will be described using Kaplan–Meier curves.

The analysis of the primary outcome (i.e., distribution of VFDs that is highly skewed) will be performed by a multivariate non-parametric test, taking into account stratification and minimization criteria used in the randomization [21]. The final test will be performed at a 0.049 two-sided alpha risk to guarantee a global 5% alpha level.

The main analysis will be performed according to the intention-to-treat principle on the full dataset, while analyses on imputed data dealing with missingness will be considered as sensitivity analyses. As a secondary analysis, a time-to-event model, where death and becoming ventilator free are competing risks, with a fixed factor corresponding to the randomization arm, centre being considered as a random factor, taking into account other prognostic factors and their interactions with the randomized treatment, will be developed.

The analysis of the effect of the intervention on secondary criteria: mortality, adverse events, and length of ventilation, will be performed using generalized linear model regression. The link function and distribution of errors will be chosen according to the nature of the response: logistic and binomial if the response is binary (mortality), identity and Gaussian if the response is considered continuous. Time-to-event censored data (becoming ventilation free or death being competing risks) will be analysed by piecewise exponential regression models. The main secondary analyses will firstly be stratified on randomization criteria without further adjustment then, after bivariate screening, the main confounders (*p* value < 0.2 or retrieved from the literature) and interactions with treatment will be tested in a multivariable model. The shape of the relationship of continuous factors will be explored by fractional polynomials. Goodness-of-fit will be verified (influential observations, R2, discrimination, calibration) and performance of the models will be assessed by Akaike information criteria.

These analyses will be performed on the intention-to-treat groups, except for safety or tolerability criteria, which will be analysed on per-protocol populations.

Analyses will be performed in the NIV or invasive ventilation subgroup (prespecified in the analysis plan) to assess the homogeneity of the difference between the two treatment groups. These subgroup analyses will be supplemented by interaction tests between the treatment and the subgroup variables using Breslow & Day tests. The results will be graphically presented using Forest plots.

### Interim analyses {21b}

No interim analyses are planned.

### Methods for additional analyses (e.g. subgroup analyses) {20b}

Analyses will be performed in the NIV and invasive ventilation subgroups, in the pneumonia subgroups (yes/no), and in the subgroups of patients who received/not corticosteroids in the last 48 h before randomization (prespecified in the analysis plan) to assess the homogeneity of the difference between the two treatment groups. These subgroup analyses will be supplemented by interaction tests between the treatment and the subgroup variables using Breslow & Day tests. The results will be graphically presented using Forest plots.

### Methods in analysis to handle protocol non-adherence and any statistical methods to handle missing data {20c}

The trial will follow the modified intent-to-treat principle as defined above, so we will not exclude data from the database in case of intervention discontinuation. We will follow the participants who discontinue the intervention during the study duration, unless the participant asked not to be followed.

### Patient and public involvement {31c}

Patients and the public were not involved in the study design. Participants will be able to have access to the findings of the study on reasonable request.

Data are available on reasonable request. The procedures carried out with the French data privacy authority (Commission Nationale de l'Informatique et des Libertés) do not provide for the transmission of the database, nor do the information and consent documents signed by the patients. Consultation by the editorial board or interested researchers of individual participant data that underlie the results reported in the article after deidentification may nevertheless be considered, subject to prior determination of the terms and conditions of such consultation and in respect for compliance with the applicable regulations.

## Oversight and monitoring

### Composition of the coordinating centre and trial steering committee {5d}

The trial will be overseen by a steering committee (principal investigator, scientific director, senior investigators, and methodologist) regarding the progression and monitoring of the study. The members of the steering committee are as follows: Alexis Ferré (coordinating investigator), Alexandre Demoule (scientific director), Thomas Similowski (senior investigator), Arnaud W. Thille (senior investigator), Armand Mekontso-Dessap (senior investigator), Philippe Aegerter (methodologist), Laure Morisset (project manager), and Virginie Chatagner (project manager).

### Composition of the data monitoring committee, its role and reporting structure {21a}

Although both strategies (corticosteroid treatment or no corticosteroids) are currently used in routine practice, a data safety monitoring board (DSMB) was involved to ensure the safety of the patients participating in this research. The members of the DSMB will not otherwise be involved in the study. They will be appointed for the duration of the study and will undertake to participate and respect the data confidentiality. The DSMB will only have an advisory role. They will meet—as required by the trial sponsor and at least once after the inclusion of the first 50 patients and once after the inclusion of the first 200 patients—to analyse tolerance and adverse events that occur during this trial and will decide what action to be taken, if any.

The members of the DSMB are as follows: Elie Azoulay, MD-PhD (ICU, Hôpital Saint-Louis, Paris, France), Muriel Fartoukh, MD-PhD (ICU, Hôpital Tenon, Paris, France), Nicolas Roche, MD-PhD (Pulmonology, Hôpital Cochin, Paris, France), and Mathieu Resche-Rigon, MD-PhD (Biostatistics, Hôpital Saint-Louis, Paris, France).

### Adverse events reporting and harms {22}

Included patients will be monitored closely for clinical (daily) and biological (as appropriate in routine practice) monitoring to ensure accurate assessment of the safety of the study. Serious adverse events (SAEs) will be collected throughout the study and during the 20 days after the last experimental treatment injection. The DSMB will be regularly informed of all SAEs.

Corticosteroids have been widely used worldwide in numerous medical indications for decades. Therefore, the adverse events related to corticosteroids are well known and expected in the vast majority of cases.

Any SAE, regardless of its causal relationship to the research (except those identified in the protocol as not requiring immediate reporting), must be reported to the sponsor.

Any SAE must be declared to the study sponsor as soon as the investigating physician becomes aware of it. The initial declaration may be followed by additional information within 8 days in the event of a fatal or life-threatening event and within 15 days in other cases. The investigator is responsible for noting and reporting all SAEs (apart from the exceptions prespecified list) that occur during the entire study, from the date of signed consent and throughout the participant’s follow-up period. Moreover, regardless of the time after the study end, any SAE that may be due to the research must be declared to the sponsor if no other cause than the research can reasonably be attributed.

Each SAE will be described on the appropriate form in an attempt to be as exhaustive as possible.

If an adverse event occurs, the investigator can either continue the assigned treatment or temporarily or permanently withdraw it. In addition, surveillance will be strengthened, and a symptomatic product can be added.

### Frequency and plans for auditing trial conduct {23}

The sponsor has scheduled regular monitoring by a dedicated research technician to ensure the proper conduct of the study. The timing of the monitoring will consist of one visit (2 days) per centre every 3 months.

### Plans for communicating important protocol amendments to relevant parties (e.g. trial participants, ethical committees) {25}

Any modifications to the protocol that impact the conduct of the study, the potential benefit to the patient, or that may affect patient safety—including changes to the study objectives, study design, patient population, sample sizes, study procedures, or significant administrative aspects—will require a formal amendment to the protocol. Such amendment will be submitted to the Comité de Protection des Personnes Ouest 6 (Brest, France).

Important protocol changes, as well as changes in eligibility criteria, outcomes, or analyses, will be communicated to the investigators, institutional ethics committee, trial participants, and trial registries.

### Dissemination plans {31a}

Findings will be published in peer-reviewed journals and presented at national and international meetings. Communications, reports, and publications of the study results will be placed under the responsibility of the principal coordinating investigator of the study and the steering committee. Reporting will follow the CONSORT statement and rules of publication will follow the international recommendations according to The Uniform Requirements for Manuscripts (International Committee of Medical Journal Editors, April 2010).

## Discussion

To date, there have been no conclusive randomized controlled trial on the benefits of corticosteroids for patients with COPD during severe acute exacerbations requiring mechanical ventilation (invasive or NIV) in the ICU [10–12]. International guidelines that recommend corticosteroids relying on a previous study with a small sample of non-ICU patients and heterogenous results [10]. Severe COPD exacerbations are still an important medical burden and the routine use of NIV in the 1990s improved survival [22]. Since then, the ICU mortality rate has remained stable (15%) [3]. Decreasing the duration of mechanical ventilation and avoiding the need for invasive mechanical ventilation are associated with better outcomes and are of high interest in the ICU. Symptomatic therapies during COPD exacerbations are limited [10]. Among the numerous factors associated with NIV failure or delayed weaning from invasive mechanical ventilation, the roles of persistent airway inflammation and bronchial spasm have been highlighted, and these could be reduced with systemic corticosteroids. However, their benefit is still a matter of debate and there is a lack of evidence [11, 12]. We expect our study to add a significant contribution regarding the benefits of corticosteroids for patients with COPD. Of note, the numerous adverse events associated with corticosteroids are not without consequences for frail patients and could impact prognosis, e.g. hyperglycaemia, intestinal haemorrhage, uncontrolled arterial hypertension, ICU-acquired weakness, and ICU-acquired infection. The study will therefore pay special attention to these adverse events.

Overall, we hope that this trial will help to revise international recommendations with a high level of evidence for patients with severe acute COPD exacerbation requiring NIV or invasive mechanical ventilation in the ICU.

## Trial status

- The current version of the protocol is Version 5.0, November, 3, 2022.
- The first patient was included in the study on October, 21, 2021.
- The recruitment period is 24 months (2 years).

## Data Availability

The investigators will make the documents and individual data strictly required for monitoring, quality control, and audit of the study available to dedicated persons, in accordance with laws and regulations in force (Articles L.1121–3 and R.5121–13 of the Code de Santé Publique – CSP, French Public Health Code). The datasets used and/or analysed during the current study will be available from the coordinating investigator on reasonable request. The procedures carried out under the French data privacy authority (Commission Nationale de l'Informatique et des Libertés) do not permit the transmission of the database, nor do the informed consent documents signed by the patients. Consultation by the editorial board or interested researchers of individual participant data that underlie the results reported in the article after deidentification may nevertheless be considered, subject to prior determination of the terms and conditions of such consultation and in respect of compliance with the applicable regulations.
